# Molecular Cloning, Heterologous Expression, and Functional Characterization of an NADPH-Cytochrome P450 Reductase Gene from *Camptotheca acuminata*, a Camptothecin-Producing Plant

**DOI:** 10.1371/journal.pone.0135397

**Published:** 2015-08-07

**Authors:** Xixing Qu, Xiang Pu, Fei Chen, Yun Yang, Lixia Yang, Guolin Zhang, Yinggang Luo

**Affiliations:** 1 Center for Natural Products Research, Chengdu Institute of Biology, Chinese Academy of Sciences, Chengdu, PR China; 2 State Key Laboratory of Bioorganic and Natural Products Chemistry, Shanghai Institute of Organic Chemistry, Chinese Academy of Sciences, Shanghai, PR China; 3 University of Chinese Academy of Sciences, Beijing, PR China; Institute of Zoology, Chinese Academy of Sciences, CHINA

## Abstract

Camptothecin (CAM), a complex pentacyclic pyrroloqinoline alkaloid, is the starting material for CAM-type drugs that are well-known antitumor plant drugs. Although many chemical and biological research efforts have been performed to produce CAM, a few attempts have been made to uncover the enzymatic mechanism involved in the biosynthesis of CAM. Enzyme-catalyzed oxidoreduction reactions are ubiquitously presented in living organisms, especially in the biosynthetic pathway of most secondary metabolites such as CAM. Due to a lack of its reduction partner, most catalytic oxidation steps involved in the biosynthesis of CAM have not been established. In the present study, an NADPH-cytochrome P450 reductase (CPR) encoding gene *CamCPR* was cloned from *Camptotheca acuminata*, a CAM-producing plant. The full length of *CamCPR* cDNA contained an open reading frame of 2127-bp nucleotides, corresponding to 708-amino acid residues. CamCPR showed 70 ~ 85% identities to other characterized plant CPRs and it was categorized to the group II of CPRs on the basis of the results of multiple sequence alignment of the N-terminal hydrophobic regions. The intact and truncate CamCPRs with N- or C-terminal His_6_-tag were heterologously overexpressed in *Escherichia coli*. The recombinant enzymes showed NADPH-dependent reductase activity toward a chemical substrate ferricyanide and a protein substrate cytochrome c. The N-terminal His_6_-tagged CamCPR showed 18- ~ 30-fold reduction activity higher than the C-terminal His_6_-tagged CamCPR, which supported a reported conclusion, i.e., the last C-terminal tryptophan of CPRs plays an important role in the discrimination between NADPH and NADH. Co-expression of CamCPR and a P450 monooxygenase, CYP73A25, a cinnamate 4-hydroxylase from cotton, and the following catalytic formation of *p*-coumaric acid suggested that CamCPR transforms electrons from NADPH to the heme center of P450 to support its oxidation reaction. Quantitative real-time PCR analysis showed that *CamCPR* was expressed in the roots, stems, and leaves of *C*. *acuminata* seedlings. The relative transcript level of *CamCPR* in leaves was 2.2-fold higher than that of roots and the stems showed 1.5-fold transcript level higher than the roots. The functional characterization of CamCPR will be helpful to disclose the mysterious mechanisms of the biosynthesis of CAM. The present study established a platform to characterize the P450 enzymes involved in the growth, development, and metabolism of eukaryotic organisms.

## Introduction

**Fig 1:**
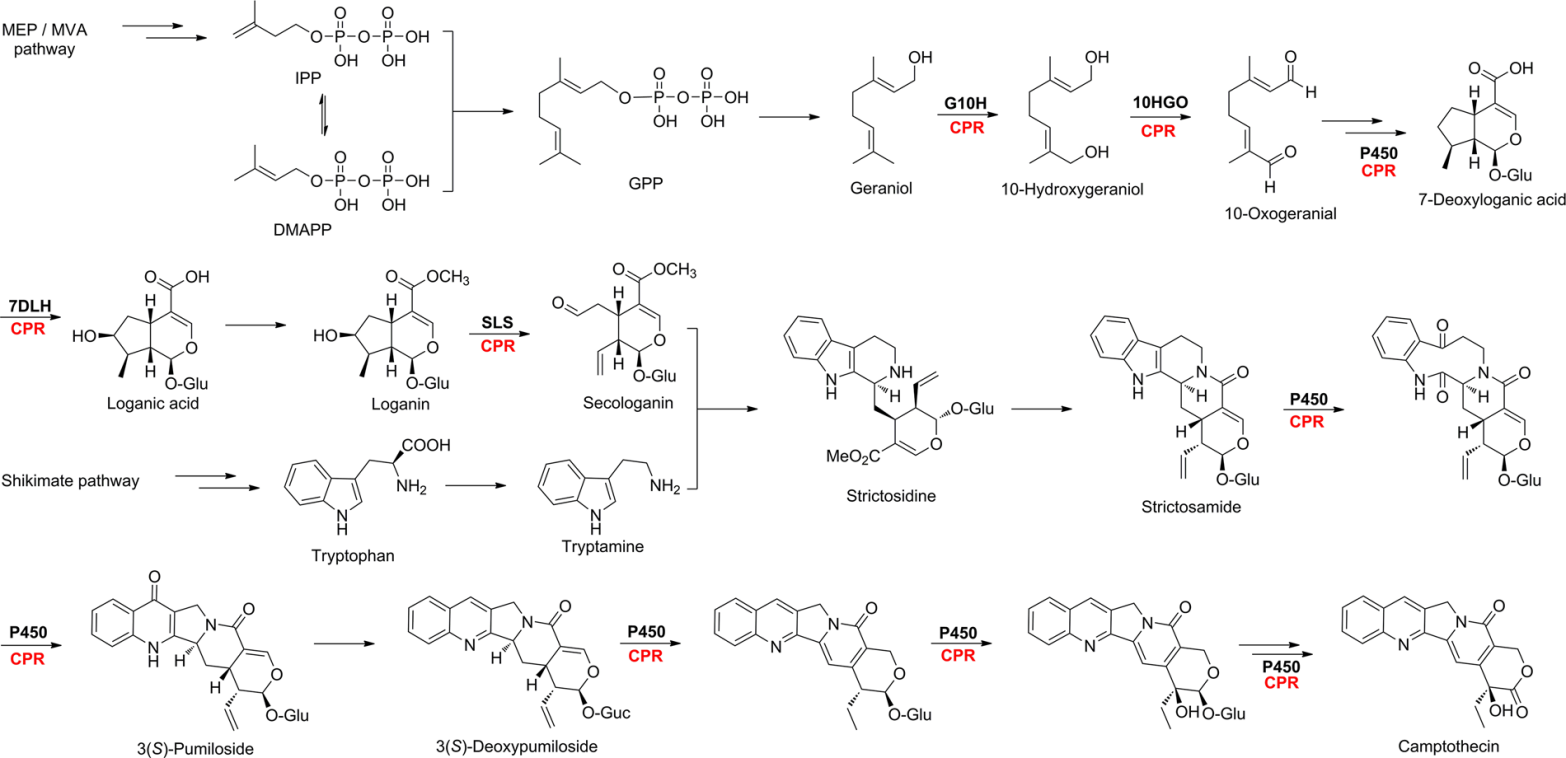
Putative biosynthetic pathway of CAM [11–20]. The terpenoid precursor secologanin was biosynthesized from MVA and / or MEP pathway through more than five steps of enzymatic oxidation. The amine precursor tryptamine was originated from shikimate pathway to tryptophan and followed by decarboxylation reaction. More than five cytochrome P450 enzymes were predicted to be involved in the conversion from strictosidine to CAM. The possible cytochrome P450 enzymes were highlighted in bold and its reduction partner CPR was in red. The other enzymes involved in the biosynthesis of CAM were omitted for clarity.

Camptothecin (CAM, Fig 1), a complex pentacyclic pyrroloqinoline alkaloid, was identified firstly from a China native tree *Camptotheca acuminata* [1]. CAM was verified to induce protein-linked DNA breakage via mammalian DNA topoisomerase I [2]. Since then CAM-type drugs, including 10-hydroxycamptothecin, topotecan, irinotecan and SN-38 were used as efficient anticancer drugs against a broad band of tumor types such as small lung cancer and refractory ovarian cancer [3,4]. These CAM-type drugs were chemically derived from CAM, although a small amount of 10-hydroxycamptothecin could be isolated from plant resources [5]. CAM itself was produced mainly by *C*. *acuminata* in China and *Nothapodytes foetida* in India [6], though several plant species of the Asterid clade, including Icacinaceae (*Pyrenacantha klaineana* and *Merrilliodendron megacrapum*), Rubiaceae (*Ophiorrhiza pumila* and *O*. *mungos*), Apocynaceae (*Ervatamia heyneana*) and Gelsemiaceae (*Mostuea brunonis*) have been reported to produce CAM [7]. In recent years, the heavy demand for CAM-type drugs has resulted in destructive harvesting of these plants in China and India. Many research efforts have been performed to uncover the mystery of the CAM biosynthesis, which will be helpful to increase the production amount of CAM via plant molecular breeding, metabolic engineering, etc. [8,9,10]. However, the knowledge of the biosynthetic machinery for CAM is yet to be fully deciphered and identified [10].

CAM was proposed to be derived from strictosidine, a common key intermediate of the monoterpenoid indole alkaloids [11] (Fig 1). Catalyzed by strictosidine synthase, strictosidine was proven to be synthesized from tryptamine and secologanin via the Pictect-Spengler condensation reaction [11] (Fig 1). Subsequent intramolecular cyclization of strictosidine yielded strictosamide, a penultimate precursor of CAM formation in *C*. *acuminata* [12] (Fig 1). Strictosamide was converted to CAM via a series of oxidation, rearrangement, cyclization, deglycosylation, dehydrogenation, and hydroxylation reactions (Fig 1). Among them, pumiloside and deoxypumiloside had been thought to be biogenetic intermediates in the formation of CAM from strictosamide [13,14] (Fig 1). Tryptamine, the amine precursor of strictosidine, was proven to be decarboxylation product of tryptophan from shikimate pathway [15] (Fig 1). Secologanin, the monoterpenoid precursor of stictosidine, was believed to be derived from geranyl diphosphate that is biosynthesized from mevalonate and/or non-mevalonate pathways, through a series of hydroxylation, epoxidation, oxidation, glycosylation, methylation, and cyclization steps [16,17,18,19,20] (Fig 1).

Most of the above mentioned reaction steps (Fig 1), including hydroxylation, oxidation, carbon-carbon bond scission, and dehydrogenation reactions, were thought to be catalyzed by various cytochrome P450 monooxygenases (P450s), one of the largest superfamily of enzymes [21]. The catalytic activities of most if not all eukaryotic P450s depend on their reduction partner, cytochrome P450 reductase (CPR, E.C.1.6.2.4). As the electrons donors, CPRs transfer two electrons from NADPH through the FAD and FMN cofactors into the central heme iron of P450s [22]. CPRs were proven to be presented in most living organisms, for instance, yeasts, plants and animals [23–30]. These CPRs were functionally characterized to donate electrons to P450s to support the oxidation reactions catalyzed by P450s [23–30]. Herein we report the molecular cloning, heterologous overexpression, and functional characterization of *CamCPR*, an NADPH-dependent CPR encoding gene from *C*. *acuminata*. The present study will facilitate the cloning and functional characterization of the P450s and thus accelerate the deciphering of CAM biosynthetic mysteries.

## Materials and Methods

### Plant materials and seedling growth

The fully matured seeds of *C*. *acuminata* were collected from matured *C*. *acuminata* trees located in the campus (104°4′12″ E, 30°37′59″ N) of Chengdu Institute of Biology of the Chinese Academy of Science, Chengdu, China. The seeds were washed with 5% Triton X-100 for 3 min, rinsed 8 ~ 10 times with sterile water, immersed in 70% EtOH for 1 min, soaked in 1% NaOCl for 3 min, and then re-rinsed 8 ~ 10 times with sterile water. The surface sterile seeds were transferred to half-strength MS medium solidified with 0.35% Phytagel (Sigma-Aldrich Co. LLC., MO) in Erlenmeyer flasks and grown in continuous darkness for 7 days at 25°C. The seedlings were transferred to a growth chamber and grown under a 16-h photoperiod provided by cool white fluorescent light (40 μmol m^-2^ s^-l^). The 20-day-seedlings were collected, frozen, and stored at -80°C.

### Total RNA isolation

The total RNA was isolated from the seedlings of *C*. *acuminata* by using the *TransZol UP* Kit (Beijing TransGen Biotech Co., Ltd., China), according to the manufacturer’s manual. Briefly, the seedlings were ground into fine powder within liquid nitrogen and the total RNA was extracted using the *TransZol UP* Kit. The RNA pellets were dissolved in diethylpyrocarbonate (DEPC)-treated water. The quality and quantity of the total RNA were determined by the ratio of OD_260_ and OD_280_ recorded from UV-1100D spectrophotometer (Shanghai Mapada Instruments Co., Ltd., China). The total RNA was stored at -80°C for further usage.

### Degenerate primer design and reverse transcription—polymerase chain reaction (RT-PCR) amplification of the core amplicon of *CamCPR*

**Fig 2:**
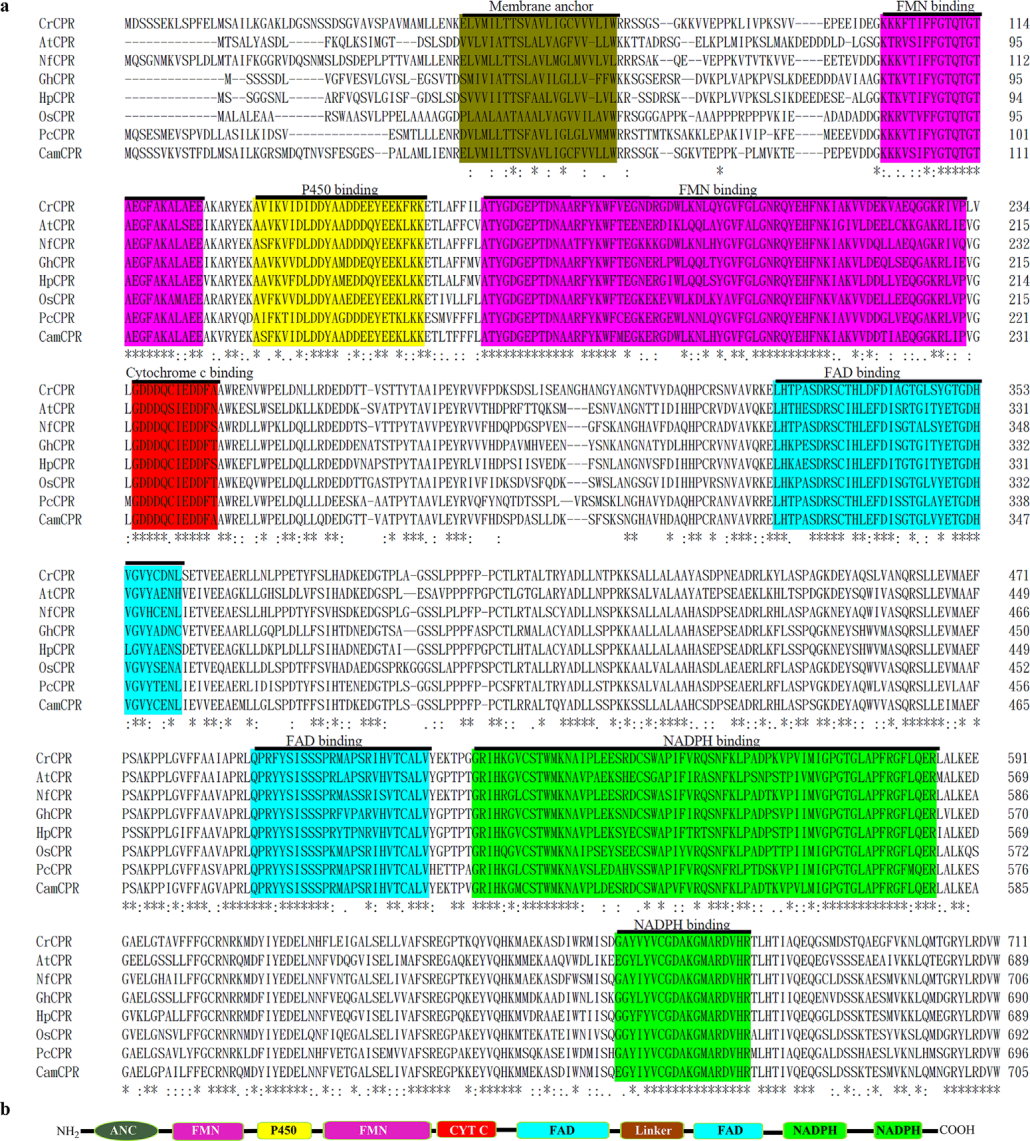
Multiple sequence alignment of the characterized plant CPRs and the deduced amino acid sequence of CamCPR using Clustal Omega multiple alignment tool (a) and schematic representation of the key domains of CamCPR (b). The conserved membrane anchor, FMN-, FAD-, P450-, cytochrome c-, and NADPH-binding domains were highlighted in different colors. The identical amino acid residues within each domain were highlighted in the same color. The cytochrome P450 reductases shown here were CrCPR from *Catharanthus roseus* (X69791), AtCPR from *Arabidopsis thaliana* (NM_118585), NfCPR from *Nothapodytes foetida* (EU604540); GhCPR from *Gossypium hirsutum* (FJ719368), HpCPR from *Hybrid poplar* (AF302496), OsCPR from *Oryza sativa* (AF302496), and PcCPR from *Petroselinum crispum* (AF024635). ANC, membrane anchor sequence; FMN, flavin mononucleotide binding domain; P450, cytochrome P450 binding domain; FAD, flavin adenine dinucleotide binding domain; NADPH, nicotine amide dinucleotide phosphate binding domain.

The amino acid residue sequences of the functionally characterized plant CPRs were retrieved from the GenBank database to perform a multiple sequences alignment by using the Clustal Omega multiple alignment tool to identify the conserved amino acid residues for a homology cloning strategy to clone the possible CPR encoding gene from *C*. *acuminata* (Fig 2). All characterized plant CPRs were proved to be membrane proteins, containing an N-terminal membrane anchor domain (Fig 2). Five highly conserved domains, including FMN-, FAD-, NADPH-, P450-, and cytochrome c-binding domains, were found in the amino acid residue sequence and structure of plant CPRs (Fig 2). Degenerate primers (Table 1) were designed on the basis of conserved amino acid residues of the FMN- and NADPH-binding sites of characterized plant CPRs (Fig 2). The primers were synthesized and purified by Sangon Biotech (Shanghai) Co., Ltd. Using the template total RNA and the degenerate primers, the *CamCPR* partial DNA was amplified by RT-PCR with One Step RNA PCR Kit (Tiangen Biotech (Beijing) Co., Ltd, China) following the standard RT-PCR program: 1 cycle of 50°C for 30 min, 1 cycle of 94°C for 2 min, 35 cycles of 94°C for 60 s, 43°C for 30 s, and 65°C for 2 min followed by a final extension at 65°C for 10 min in a thermal cycler (Eppendorf AG, Hamburg, Germany). The amplified PCR product was gel-purified and ligated into the pGM-T vector (Tiangen Biotech (Beijing) Co., Ltd, China). The constructs were transformed into *E*. *coli* DH5α competent cells and sequenced in both directions in Sangon Biotech (Shanghai) Co., Ltd. The nucleotide sequence was analyzed by using the similarity search BLAST program.

**Table 1:** List of primers used in the study.

Primer code	Sequence (5′-3′)	Direction	Application
CamCPR-F	CA(A/G)AC(C/T)GGIACIGC(C/T)GA(A/G) G	Forward	Degenerate primers
CamCPR-R	TT(A/G)GC(A/G)TCACC(A/G)CAIACATA	Reverse	
AAP	GGCCACGCGTCGACTAGTACGGGIIGGGIIGGGIIG	Forward	5′-RACE
5GSP1	TCATACTGCCTGTTGCCGAGAC	Reverse	
5GSP2	CCATCTCCGTATGTAGCTAAGAAG	Reverse	
3GSP1	TACCGTCGTTCCACTAGTGATTT	Forward	3′-RACE
3RACE Outer Primer	TACCGTCGTTCCACTAGTGATTT	Reverse	
CamCPR-Full-F	CGGTAAGATGCAATCGAGTTCG	Forward	Full length cloning
CamCPR-Full-R	GAATTGGCAGCGAAGGTGAGTA	Reverse	
His_6_-CamCPR-F	GGAATTCCATATGCAATCGAGTTCGG	Forward	Overexpression
His_6_-tCamCPR-F	GGAATTCCATATGTCGTCAGGAAAGTC	Forward	
His_6_-CamCPR-R	CCGCTCGAGTCACCACACATCACGC	Reverse	
CamCPR-His_6_-F	CATGCCATGGCATCGAGTTCGGTTAAG	Forward	
tCamCPR-His_6_-F	GGAATTCCATATGTCGTCAGGAAAGTC	Forward	
CamCPR-His_6_-R	CCCAAGCTTCCACACATCACGCAAATACC	Reverse	
rtCamCPR-F	TGAAACTGGTGCACTCTCTGAG	Forward	Quantitative real-time PCR analysis
rtCamCPR-R	CAGTCTTGGAGCTGTCTAGAGATC	Reverse	
rtActin-F	TTCCTCATGCCATCCTCCGTCTT	Forward	
rtActin-R	CAGCGATACCTGAGAACATAGTGG	Reverse	

### Rapid amplification of cDNA ends (RACE) of *CamCPR*

To get the complete open reading frame of *CamCPR* by using RACE methods, gene-specific primers (GSPs, Table 1) were designed on the basis of the nucleotide sequence of the core amplicon of *CamCPR*. The 5′- and 3′-ends of *CamCPR* were obtained by using 5′-RACE system for Rapid Amplification of cDNA Ends, Version 2.0 (Invitrogen, Shanghai) and 3′-Full RACE core set with PrimeScript RTase (Takara Biotechnology (Dalian) Co., Ltd, China), respectively, according to the manufacture’s corresponding instructions. Briefly, 5GSP1 was used as primer and the total RNA as template to get the first strand cDNA through S. N. A. P. purification. The purified cDNA was tailored with dCTP catalyzed by terminal transferase TdT. PCR amplification of the dC-tailored cDNA was performed using 5GSP2 and AAP as primers to afford the 5′-end of *CamCPR*, following the cycling conditions: 1 cycle of 94°C for 3 min, 35 cycles of 94°C for 30 s, 50°C for 1 min, 72°C for 2 min followed by a final extension at 72°C for 10 min. Meanwhile, 3′-RACE adaptor was used as primer and the total RNA as template to afford the first strand cDNA of the 3′-end of *CamCPR*. Subsequent PCR amplification was performed using 3GSP1 and 3′-RACE outer primer to obtain the 3′-end of *CamCPR*, following the cycling conditions: 1 cycle of 94°C for 3 min, 35 cycles of 94°C for 30 s, 50°C for 1 min, 72°C for 2 min followed by a final extension at 72°C for 10 min. The amplification products of both 5′- and 3′-RACE PCR were gel-purified, ligated into the pGM-T vector, and transformed into *E*. *coli* DH5α competent cells, respectively, following the procedure mentioned above. The nucleotide sequences of the 5′- and 3′-ends of *CamCPR* were sequenced in Sangon Biotech (Shanghai) Co., Ltd.

### *CamCPR* full-length cDNA cloning

The full-length cDNA of *CamCPR* was generated on the basis of the above mentioned sequences of the core amplicon fragments, 5′- and 3′-RACE products. Two specific primers, CamCPR-Full-F and CamCPR-Full-R (Table 1), were designed and synthesized to clone the full-length of *CamCPR* from the total RNA of *C*. *acuminata*. Subsequently RT-PCR amplification using the above mentioned primers and total RNA as template afforded the desired PCR products. The RT-PCR conditions were 1 cycle of 50°C for 30 min, 35 cycles of 94°C for 30 s, 55°C for 30 s, and 65°C for 3 min followed by a final extension at 65°C for 10 min. The RT-PCR products were gel-purified, ligated into the pGM-T vector, and transformed into *E*. *coli* DH5α competent cells, following the procedure mentioned above. The nucleotide sequences of the full length of *CamCPR* were sequenced in Sangon Biotech (Shanghai) Co., Ltd.

### Bioinformatics analyses of *CamCPR* and its encoding protein

The full length nucleotide sequence of *CamCPR* was set as a query to search the nucleotide database of NCBI (http://www.ncbi.nlm.nih.gov/) by using BLAST program. The open reading frame (ORF) of *CamCPR* was predicted by using Translate tool (http://www.expasy.ch/tools/dna.html/). The properties of the deduced amino acid sequences of *CamCPR* were estimated by using the ExPASy ProtParam tool (http://www.expasy.ch/tools/protparam.html/). The transmembrane domain and N-terminal signal peptide of CamCPR were predicted by using TMHMM (http://www.cbs.dtu.dk/services/TMHMM/) and SignalP (http://www.cbs.dtu.dk/services/SignalP/), respectively. The subcellular location of CamCPR was predicted by using WoLF PSORT (http://wolfpsort.org/). Clustal Omega (http://www.ebi.ac.uk/Tools/msa/clustalo/) was used for multiple sequence alignment. Structurally and functionally important regions were identified in the deduced amino acid sequence of CamCPR by Conseq services (http://consurf.tau.ac.il/). To assess the evolutionary relationships between the CamCPR and other CPR homologs from different plants species, the CamCPR was set as a query to search the database of NCBI by using BLASTp searches and the amino acid sequences of characterized CPRs were retrieved from NCBI. Then the amino acid residue sequences of CPRs were aligned using the ClustalW program (http://www.ebi.ac.uk). A phylogenetic tree was constructed by neighbour-joining method using MEGA 5 software [31]. Bootstrap analysis with 1,000 replicates was also conducted in order to obtain confidence levels for the branches.

### Heterologous overexpression of CamCPR in *E*. *coli*

Different forward and reverse primers (Table 1) with various endonucleases restriction sites were designed and synthesized to amplify the expected nucleotide sequence of *CamCPR* by PCR using a HiFi Taq DNA polymerase (Sangon Biotech (Shanghai) Co., Ltd., China). It should be noted that two forward primers (Table 1), His_6_-tCamCPR-F for N-terimal His_6_-tag and tCamCPR-His_6_-F for C-terminal His_6_-tag, were designed to truncate the N-terminal 69 amino acid residues of CamCPR to afford the truncated CamCPRs (tCamCPR), according to a previous report [18]. The PCR conditions used were: 1 cycle of 94°C for 3 min, 35 cycles of 94°C for 30 s, 55°C for 30 s, and 72°C for 2 min followed by a final extension at 72°C for 10 min. The PCR products were gel-purified, digested with the corresponding endonucleases, and subcloned into the corresponding vectors (pET-28a for intact CamCPR and pET-30a for tCamCPR) digested with the same endonucleases to afford the expression constructs. The *E*. *coli* BL21(DE3) component cells were transformed with the expression constructs to afford the recombinant strains.

For protein expression, a single colony of recombinant strain was inoculated into 5 mL of Luria–Bertani (LB) broth containing 50 μg/mL of kanamycin and incubated overnight at 37°C, 200 rpm in a shaking incubator. An aliquot culture (1 mL) was inoculated into 500 mL of Terrific Broth (TB) medium supplemented with 50 μg/mL of kanamycin and incubated at 37°C and 200 rpm. When the optical density (A_600 nm_) of the culture reached 0.8, the destination protein overexpression was induced by adding 1mM of isopropyl β-D-1-thiogalactopyranoside (IPTG; Sangon, Shanghai, China) into the culture. The culture was incubated at 28°C for another 16 h. Cells were harvested by centrifugation at 4000 rpm for 15 min at 4°C, washed twice with MOPS buffer (100 mM MOPS, 10% glycerol, 0.2 mM DTT, 1 mM EDTA, adjusted to pH 7.3 with NaOH, 4°C), re-suspended in the same buffer containing 1 mg/mL lysozyme and 1 mM phenylmethylsulfonyl fluoride (PMSF), and kept at 4°C for 30 min. The suspended cells were sonicated on ice-bath followed by centrifugation at 15,000 rpm for 30 min at 4°C. For purification of the membrane proteins, one more step was applied before the affinity chromatography. The supernatant was mixed with Triton X-100 (10%) to solubilize the membrane proteins with stirring gently on ice for 2 h. The mixture was centrifuged at 15,000 rpm for 30 min at 4°C and the resulting supernatant was incubated with nickel-nitrilotriacetic acid resin (Sangon) for 30 min. The mixtures of membrane protein or soluble protein and resin were loaded on to a gravity flow column and then eluted with MOPS buffer containing different concentration of imidazole (10, 50, and 250 mM). Purified protein was desalted by dialysis membranes (Sangon) with Tris-HCl buffer (50 mM Tris-HCl, pH 7.4). The purified protein was stored with Tris-HCl buffer containing 20% glycerol at -20°C. The purified protein samples were analyzed on 10% SDS-PAGE and their concentrations were estimated by using the ε _280 nm_ calculated from ExPASY ProParam as follows: His_6_-CamCPR and CamCPR-His_6_ (ε _280 nm_ = 90,020 M^-1^ cm^-1^), His_6_-tCamCPR and tCamCPR-His_6_ (ε _280 nm_ = 84,395 M^-1^ cm^-1^).

For the bacterial co-expression system used for P450 monooxygenase activity assay, the tCamCPR was inserted into *Nde*I / *Xho*I sites of pETDUET-1 (Novagen, Madison, USA), and the native ORF of CYP73A25 was inserted into the *Sal*I / *Not*I sites of either empty pETDUET-1 or pETDUET-1 harboring tCamCPR. The constructs were transferred into *E*. *coli* BL21(DE3) component cells. The recombinant strain was overexpressed in TB medium with 100 μg/mL of ampicillin as described above. The bacterial cells were collected by centrifugation at 4000 rpm for 5 min at 4°C, washed twice with chill MOPS buffer, re-suspended in the same buffer containing 1 mg/mL lysozyme, and kept at 4°C for 30 min. After sonication on ice-bath, the cell lysate was centrifuged at 12,000 rpm for 10 min, and the supernatant was subjected to P450 activity assay. Overexpression of CYP73A25 and tCamCPR protein was confirmed by SDS-PAGE analysis. The reduction activity of tCamCPR was validated by cytochrome c reducing activity assay.

### Enzymatic activity of CamCPR toward protein substrate

Cytochrome c reducing activity of CamCPR was assayed as described previously with minor modifications [32]. The reduction was monitored by the increase of absorbance at 550 nm, at 25°C. Briefly, 500 μL of the reaction mixture contained 25 μM cytochrome c in 50 mM Tris-HCl buffer (pH 7.4) and 1 μg protein. The reaction was initiated by adding 25 μM NADPH. The time dependent absorption change at 550 nm was recorded on a UV-1100D spectrophotometer. An absorption coefficient of 21 mM^-1^ cm^-1^ for equine heart cytochrome c was used for quantification. To determine kinetic parameters for cytochrome c, 100 μM NADPH was added to the reaction mixtures containing various concentrations of cytochrome c. The kinetic parameters for NADPH were measured by using 75 μM cytochrome c with varying NADPH concentrations. The kinetic constants *K*_m_ and *V*_max_ were calculated with nonlinear regression analysis using Origin 8.0 software (OriginLab Corporation, Northampton, USA). All concentration points were assayed in duplicate.

### Enzymatic activity of CamCPR toward chemical substrate

According to the reported procedure [23], the ability of CamCPR to reduce chemical substrate ferricyanide was determined using 6.25 ~ 200 μM of substrate and 10 nM CamCPR in 50 mM phosphate buffer (pH 8.0). The change in absorbance at 420 nm for ferricyanide substrate was measured following the addition of varying NADPH concentrations. The reduction rates were calculated using extinction coefficients of 1.02 mM^-1^cm^-1^ for ferricyanide. The kinetic parameters were determined as mentioned above.

### CamCPR supported heterogenous P450 monooxygenase activity

A functionally characterized P450 monooxygenase from cotton, CYP73A25 [24], was used to validate the function of CamCPR as a cytochrome P450 reductase. Overexpression of CYP73A25 and tCamCPR were performed as mentioned above. The cinnamic acid 4-hydroxylase activity was assayed in 50 mM phosphate buffer (pH 7.4) containing 1 mM *trans*-cinnamic acid, 1 mM glucose-6-phosphate and 1 mM glucose-6-phosphate dehydrogenase. The reaction was initiated by adding 2 mM NADPH and incubated at 30°C for 2 h. To quench the reaction, an equal volume of chill CH_3_OH was added to the reaction mixture. The formation of *p*-coumaric acid was determined by an HPLC equipped with an Altima C_18_ analytical column (250 mm×4.6 mm, 5 μm). The mobile phase consisted of buffer A (CH_3_OH) and buffer B (H_2_O), followed a linear gradient from buffer A/buffer B (20:80) to 95% buffer A over 22 min, from 95 to 20% buffer A over 2 min and continued at 20% buffer A for an additional 2 min, at a flow rate of 1 mL/min at 35°C, and monitored by a DAD detector.

### Molecular modelling and 3-D structure prediction of CamCPR

The crystal structure of *Rattus norvagicus* CPR (RnCPR, PDB ID: c1j9zB) was resolved in high resolution. Here it was used as template to build the 3-D structure of CamCPR by using the toolkit from PHYRE2 server (Protein Homology/analogY Recognition Engine V 2.0, http://www.sbg.bio.ic.ac.uk/phyre2/html/). Based on the 3-D structure of RnCPR, the structurally, evolutionarily and functionally important regions of CamCPR were identified by using ConSurf software (http://consurf.tau.ac.il/) from the deduced amino acid residue sequence of CamCPR. The stereo-chemical and topological analyses of the modelled CamCPR were performed by using Ramachandran plotting obtained from Procheck module of the SAVES server (http://services.mbi.ucla.edu/SAVES/) and PDBSum (http://www.ebi.ac.uk/thornton-srv/databases/pdbsum/Generate.html), respectively.

### Quantitative real-time PCR

The *C*. *acuminata* seedlings were grown under identical conditions and three different individuals were set as biological replicates. A pair of specific primers, rtCamCPR-F and rtCamCPR-R, was designed for real-time PCR of *CamCPR* (Table 1), according to the instructions of real-time PCR system. Also as a control, a pair of primers for the reference gene actin was synthesized too (Table 1). To quantitate the tissue-specific expression of *CamCPR*, the fresh *C*. *acuminata* seedlings of 20 days were collected to extract the total RNA using the *TransZol UP* Kit as described before. According to the manufacturer’s instructions (Tiangen), the total RNA solution was pre-incubated at 72°C for 5 min to reduce secondary structures when used. Using the TIANScript cDNA synthesis kit (Tiangen), the cDNA (in a volume of 20 μL) was synthesized from 2 μg of the pre-incubated total RNA and the oligo (dT) primer. The above prepared cDNA of each sample was used as template for SYBR green PCR amplification to quantitate the tissue-specific expression of the target genes. The SYBR green PCR amplification was performed using the PIKOREAL24 real-time PCR System (Thermo Scientific, USA) according to the manufacturer’s instructions. Briefly, a total volume of 25 μL SYBR green PCR amplification solution contained 0.25 μL cDNA template, 0.2 μM of the primers, and 12.5 μL of AceQ qPCR SYBR Green Master Mix (Vazyme Biotech Co., Ltd., Nanjing, China). The PCR amplification was performed under the following cycling conditions: 1 cycle of 95°C for 5 min; 60 cycles of 95°C for 10 s, 58°C for 30 s, 65°C for 30 s. Each sample and the negative control without template were conducted in triplicate. To test for primer dimers and unspecific products, the melting curves of the PCR products were recorded from 60°C to 95°C on the PIKOREAL24 real-time PCR System after the PCR amplification. The real-time PCR amplification curves and the following PCR products melting curves indicated that the primers were specific for *CamCPR*. The relative transcript levels of *CamCPR* in different tissues were calculated using the corresponding transcript levels of the reference gene encoding actin in the same tissue as control. And then the mRNA amount of *CamCPR* in roots was set as control to compare the expression levels of *CamCPR* in different tissues.

## Results

### Molecular cloning of the full length cDNA encoding CamCPR

A 1673-bp fragment of the *CamCPR* gene was obtained from RT-PCR using the above mentioned primers with total RNA as template. Subsequent 5´- and 3´- flanking regions were obtained by using RACE strategy. An 865- and a 659-bp fragment, respectively, were obtained from 5´- and 3´-RACE experiments. By overlapping the nucleotide sequences of the three fragments, the full-length sequence of *CamCPR* was generated, which was confirmed by molecular cloning and subsequent DNA sequencing.

The full nucleotide sequence of *CamCPR* was 2606 bp and contained a 2127-bp ORF encoding for a 708-amino acid protein (Fig 2). The ORF was flanked by a 135-bp 5′-untranslated region (UTR) and a 344-bp 3′-UTR. The nucleotide sequence of the full-length cDNA of *CamCPR* was deposited in NCBI GenBank under the accession number KP162177.

### Bioinformatics properties of CamCPR

Using the ExPASy online tools, the theoretical isoelectric point of the deduced CamCPR was predicted to be 5.3. The molecular weight of CamCPR was 78.8 kDa. The deduced CamCPR contained the hallmarks of plant CPRs, including the membrane anchor, FMN-, FAD-, P450-, cytochrome c-, and NADPH-binding domains (Fig 2). A hydrophobic transmembrane region consisting of 19 amino acid residues was predicted at the N-terminus of CamCPR, which may function as an anchor involved in its location on the membrane of the endoplasmic reticulum. No obvious signal peptide cleavage site was predicted in CamCPR. Secondary structure prediction indicated that CamCPR consisted of α-helixes (35.77%), β-sheets (10.11%), and loops (54.12%).

**Fig 3:**
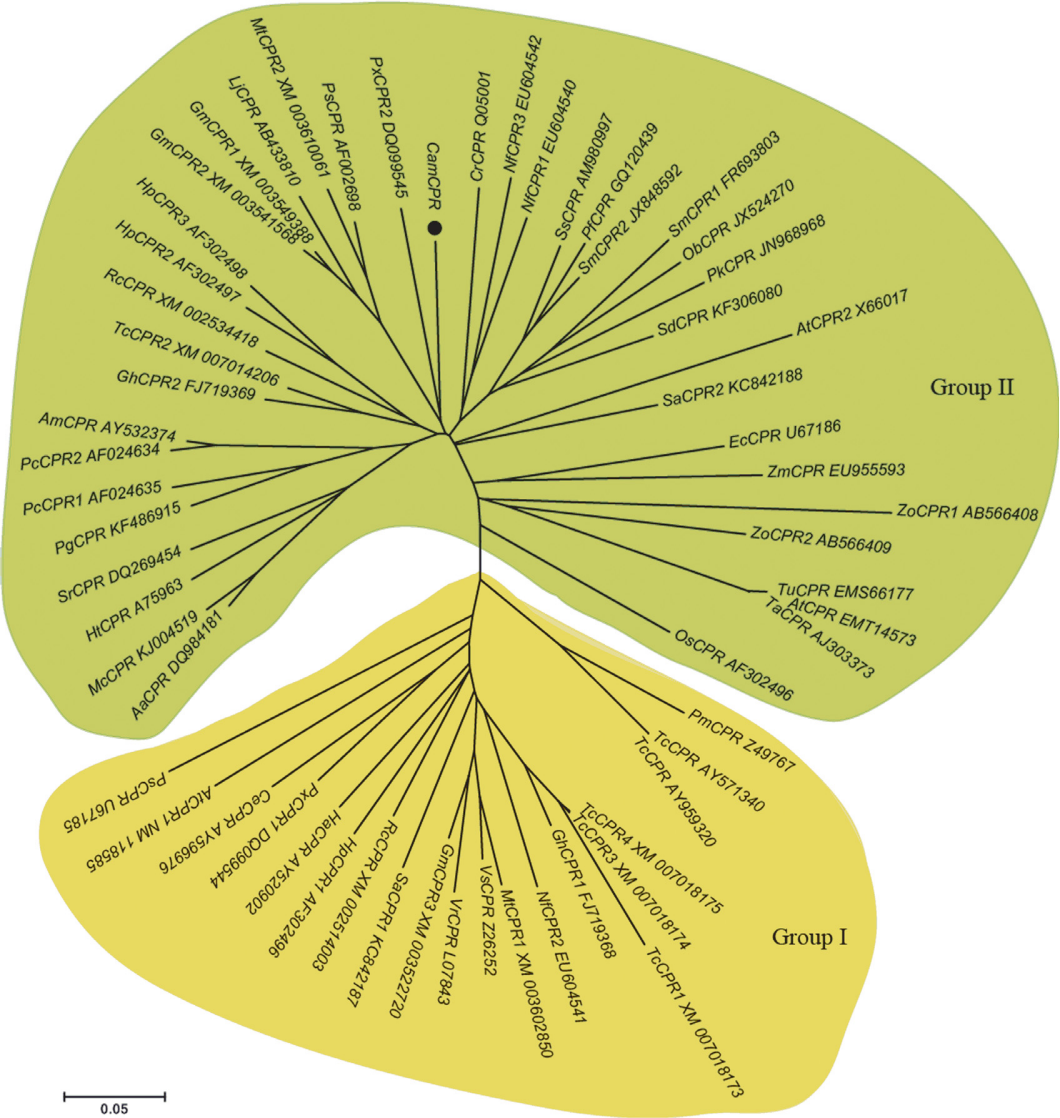
Phylogenetic tree of the cytochrome P450 reductase family members. Homologous sequences were obtained using the National Center for Biotechnology Information search engine (http://www.ncbi.nim.bih.gov/). The GenBank accession numbers for the sequences were as follows: CrCPR (*Catharanthus roseus*, X69791); AtCPR1 (*Arabidopsis thaliana*, NM_118585); AtCPR2 (*Arabidopsis thaliana*, X66017); NfCPR1 (*Nothapodytes foetida*, EU604540); NfCPR2 (*Nothapodytes foetida*, EU604541); NfCPR3 (*Nothapodytes foetida*, EU604542); GhCPR1 (*Gossypium hirsutum*, FJ719368); GhCPR2 (*Gossypium hirsutum*, FJ719369); HpCPR1 (*Hybrid poplar*, AF302496); HpCPR2 (*Hybrid poplar*, AF302497); HpCPR3 (*Hybrid poplar*, AF302498); OsCPR (*Oryza sativa Japonica*, AF302496); PcCPR1 (*Petroselinum crispum*, AF024634); PcCPR2 (*Petroselinum crispum*, AF024635); EcCPR (*Eschscholzia californica*, U67186); HtCPR (*Helianthus tuberosus*, A75963); VrCPR (*Vigna radiate*, L07843); PsmCPR (*Papaver somniferum*, U67185); PstCPR (*Pisum sativum*, AF002698); VsCPR (*Vicia sativa*, Z26252); AtCPR (*Aegilops tauschii*, EMT14573); AmCPR (*Ammi majus*, AY532374); AaCPR (*Artemisia annua*, DQ984181); CeCPR (*Centaurium erythraea*, AY596976); GmCPR1 (*Glycine max*, XM_003549388); GmCPR2 (*Glycine max*, XM_003541568); GmCPR3 (*Glycine max*, XM_003522720); HaCPR (*Hypericum androsaemum*, AY520902); LjCPR (*Lotus japonicas*, AB433810); McCPR (*Matricaria chamomilla*, KJ004519); MtCPR1 (*Medicago truncatula*, XM_003602850); MtCPR2 (*Medicago truncatula*, XM_003610061); ObCPR (*Ocimum basilicum*, JX524270); PgCPR (*Panax ginseng*, KF486915); PfCPR (*Perilla frutescens*, GQ120439); PxCPR1 (*Petunia x hybrid*, DQ099544); PxCPR2 (*Petunia x hybrid*, DQ099545); PkCPR (*Picrorhiza kurrooa*, JN968968); PmCPR (*Pseudotsuga menziesii*, Z49767); RcCPR1 (*Ricinus communis*, XM_002514003); RcCPR2 (*Ricinus communis*, XM_002534418); SmCPR1 (*Salvia miltiorrhiza*, FR693803); SmCPR2 (*Salvia miltiorrhiza*, JX848592); SaCPR1 (*Santalum album*, KC842187); SaCPR2 (*Santalum album*, KC842188); SdCPR (*Scoparia dulcis*, KF306080); SsCPR (*Solenostemon scutellarioides*, AM980997); SrCPR (*Stevia rebaudiana*, DQ269454); TchCPR (*Taxus chinensis*, AY959320); TcsCPR (*Taxus cuspidate*, AY571340); TcCPR1 (*Theobroma cacao*, XM_007018173); TcCPR2 (*Theobroma cacao*, XM_007014206); TcCPR3 (*Theobroma cacao*, XM_007018174); TcCPR4 (*Theobroma cacao*, XM_007018175); TaCPR (*Triticum aestivum*, AJ303373); TuCPR (*Triticum urartu*, EMS66177); ZmCPR (*Zea mays*, EU955593); ZoCPR1 (*Zingiber officinale*, AB566408); ZoCPR2 (*Zingiber officinale*, AB566409); Phylogenetic analysis was performed using the MEGA package and neighbor-joining program (http://www.megasoftware.net). The scale bar indicates the phylogenetic distance calculated according to the number of differences.

Similarity search showed that CamCPR shares 70 ~ 85% identities with characterized plant CPRs. To gain insights into the evolutionary relationship among CPRs from different plants, 50 CPRs from 33 dicotyls and 9 CPRs from 6 monocotyls were selected from NCBI database to align with CamCPR. A phylogenetic tree was generated by neighbor-joining method using MEGA 5 software (Fig 3). Plant CPRs were classified into groups I and II, based on the N-terminal hydrophobic regions [25]. CamCPR was phylogenetically grouped with AtCPR2, CrCPR and NfCPR1 of group II, which suggested a close evolutionary relationship within these enzymes.

### Heterologous overexpression and catalytic parameters of recombinant CamCPR

**Fig 4:**
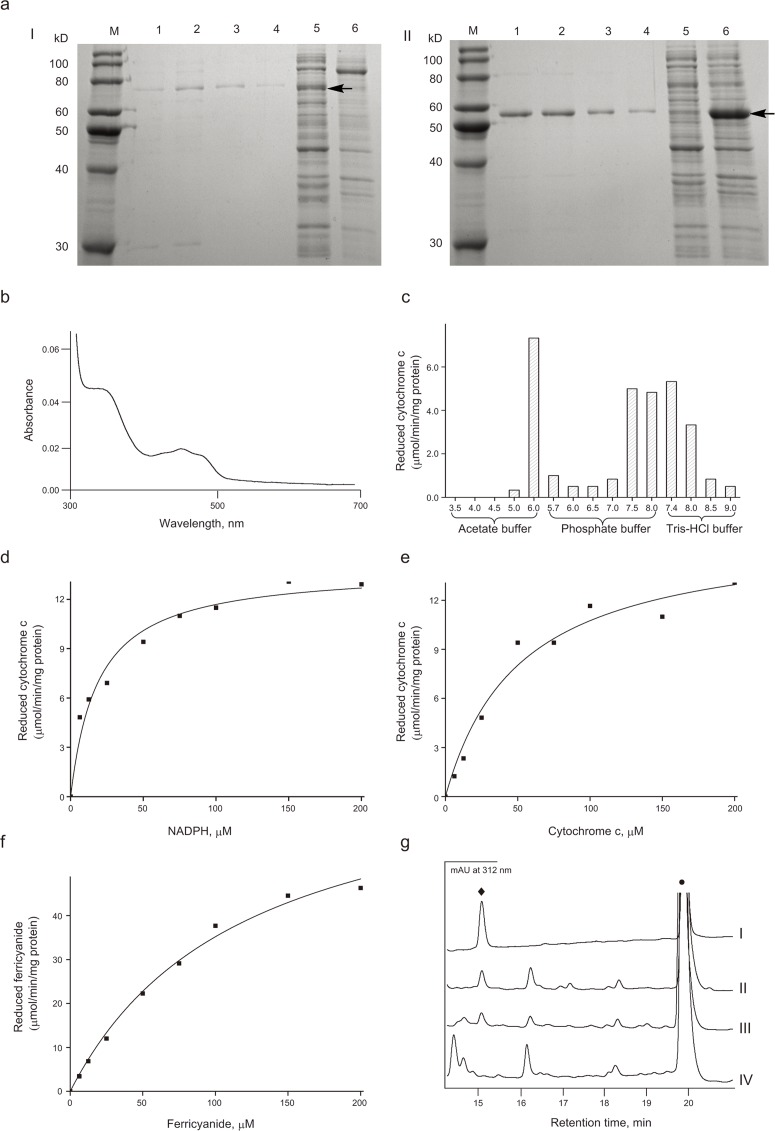
Overexpression, purification, and characterization of recombinant CamCPR. a, SDS-PAGE analyses of CamCPR (I) and tCamCPR (II). M, protein ruler; Lanes 1–4, purified enzyme; Lane 5 in I, cell lysate; Lane 5 in II, whole cell; Lane 6 in I, whole cell; Lane 6 in II, cell lysate. The target band was indicated with an arrowhead. b, the UV spectrum of tCamCPR was measured in 50 mM tris-HCl buffer (pH 7.4). c, the effects of different buffers with various pH on the cytochrome c reducing activity of tCamCPR. d–f, the steady-state kinetic constants of recombinant CamCPR. g, HPLC-DAD analyses of the reaction mixture of CamCPR supported cinnamic acid 4-hydrxoylase activity. Panel I, the authentic *p*-coumaric acid (♦) and *trans*-cinnamic acid (•); The HPLC traces of the whole reaction containing the cell lysates (panel II), the cells (panel III), and the boiled cells (panel IV) of the recombinant tCamCPR and CYP73A25 as catalyst.

According to the previous report on CrCPR from *C*. *roseus* [18], the N-terminal 69 amino acid residues of CamCPR were truncated to afford tCamCPR. The full-length ORF of CamCPR and tCamCPR were subcloned into pET-28a or pET-30a vectors and heterologously overexpressed in *E*. *coli* BL21(DE3) as a fusion protein with N- or C-terminal His_6_-tag (Fig 4a). Similar to other plant CPRs [24], the recombinant tCamCPR showed characteristic absorbance bands of a flavoprotein (Fig 4b). The reaction buffers played an important role in the cytochrome c reducing activities catalyzed with CamCPR (Fig 4c). The CamCPR showed the highest reduction activity towards cytochrome c when the reduction assay was performed in the acetate buffer with pH = 6.0. However no reduction activity was observed when it was assayed in the same acetate buffer system with pH < 4.5. Meanwhile when the reduction assays were performed in the phosphate buffer system with pH = 6.0, the CamCPR showed weaker reduction activity. However, the reduction activity of CamCPR showed constant between pH = 7.4–8.0 whether the reaction buffer is phosphate or tris-HCl buffer system. The following assays were performed in 50 mM tris-HCl buffer at pH 7.4, although the optimal cytochrome c reducing activity was found in acetate buffer at pH 6.0 (Fig 4c).

The recombinant CamCPRs were assayed for NADPH- or NADH- dependent cytochrome c reduction activities, respectively (Table 2). NADPH was efficiently served as the electron donor and NADH could not be recognized by CamCPR, which means that the activity of CamCPR is dependent on NADPH (Table 2). However, the requirement of CPRs for electron acceptors was relatively less specific. Cytochrome c and ferricyanide [K_3_Fe(CN)_6_] can serve as the electron acceptors (Fig 4e and 4f). The kinetic parameters *K*_m_ and *V*_max_ of tCamCPR for NADPH, cytochrome c, and K_3_Fe(CN)_6_ were determined, respectively. The *K*_m_ and *V*_max_ of tCamCPR were 18.7 ± 3.6 μM and 13.8 ± 0.6 μmol/min/mg protein for NADPH (Fig 4d), 51.4 ± 12.6 μM and 16.3 ± 1.5 μmol/min/mg protein for cytochrome c (Fig 4e), and 119.3 ± 17.2 μM and 77.2 ± 4.7 μmol/min/mg protein for K_3_Fe(CN)_6_ (Fig 4f).

**Table 2:** Specific activities of various CamCPRs toward cytochrome c (25 μM), in the presence of 25 μM of NADPH or NADH. Values are presented as mean ± SE. ND, not detected.

	Specific activity (μM/min/mg protein)	
	NADPH	NADH
His_6_-CamCPR	1.071±0.038	ND
His_6_-tCamCPR	3.258±0.021	ND
CamCPR-His_6_	0.038±0.006	ND
tCamCPR-His_6_	0.185±0.011	ND

### CamCPR supported heterogenous P450 monooxygenase activity

The tCamCPR was inserted into pETDUET-1, followed by the insertion of the whole ORF of CYP73A25 from the plasmid pETDUET-1-CYP73A25-GhCPR [24]. The construct was overexpressed in *E*. *coli* BL21(DE3), induced by IPTG. The cell lysate was incubated with *trans*-cinnamic acid, the native substrate of CYP73A25. Comparison the HPLC-DAD traces of the whole reaction with cell lysate as catalyst (panel II, Fig 4g) and the whole reaction with whole cell as catalyst (panel III, Fig 4g) with that of the authentic standards (panel I, Fig 4g) and the whole reaction with boiled cells as control (panel IV, Fig 4g) demonstrated the formation of *p*-coumaric acid, the desired product of cinnamate 4-hydroxylase, which confirmed that tCamCPR functionally supports CYP73A25 to convert *trans*-cinnamic acid to form *p*-coumaric acid, i.e., 4-hydroxycinnamic acid.

### Prediction of three-dimensional structure of CamCPR

**Fig 5:**
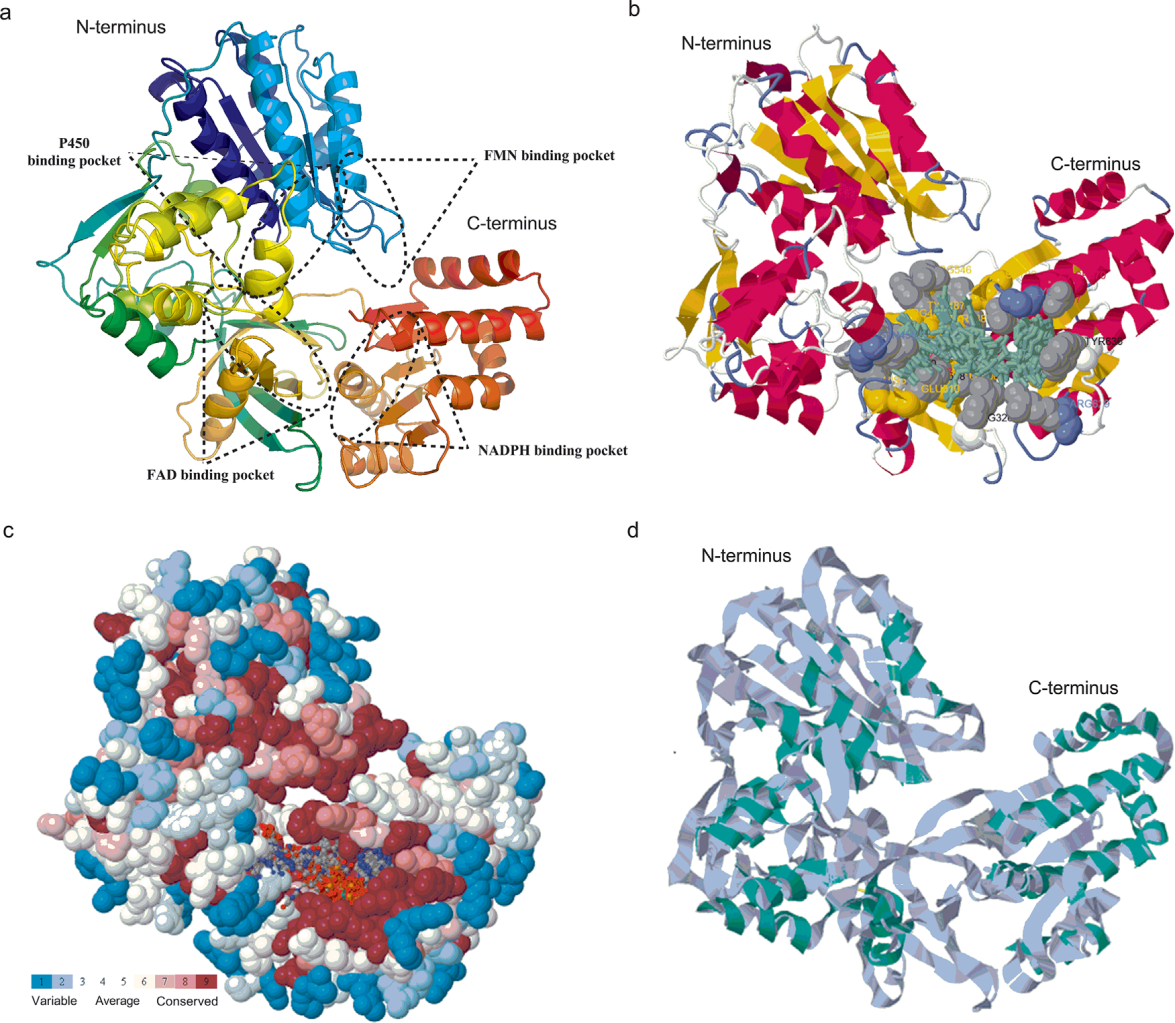
The predicted 3-D structure of CamCPR. a, schematic FMN-, FAD-, NADPH-, and P450-binding domains of CamCPR, built by the toolkit from PHYRE2 server; b, the amino acid residues involved in the binding of ligands (highlighted in greenish) such as FAD and NADPH, predicted by the 3DLigandSite tool; c, functional and structural residues of CamCPR with high scores were highlighted in red; and d, superimposition of CamCPR with the template RnCPR.

Based on the crystal structure of RnCPR (PDB ID: c1j9zB), the 3-D structure of CamCPR was predicted and constructed using different bioinformatics software (Fig 5). The presence of the conserved FMN-, FAD-, NADPH-, and P450-binding domains was also deduced from the predicted 3-D structure of CamCPR (Fig 5a). As depicted in Fig 5b, the amino acid residues involved in the binding of ligands such as FAD and NADPH were also predicted using the 3DLigandSite tool. Analyses of the evolutionary conservation of CamCPR surface amino acids indicated that many amino acid residues were conserved in CPRs. The amino acid residues with high scores were highlighted in red and were found to be the functional and structural residues of CamCPR by the bioinformatics tool of ConSeq servers (Fig 5c). Superimposition of the 3-D structures of CamCPR with the template RnCPR showed that all major domains aligned at same coordinates (Fig 5d), which indicated that CPRs were highly conserved reductases in eukaryotic organisms. The predicted structure of CamCPR was further refined by employing the bioinformatics tools from KoBaMIN web server and then the stereo-chemical qualities of the energy refined model of CamCPR was validated by the bioinformatics tools of the PROCHECK server. The results using Ramachandran plotting analysis showed that 91.6% amino acid residues of CamCPR located in the most favourable region, 6.1% residues in the additional allowed region, 1.5% in the generously allowed region, and 0.7% in the disallowed region. The relatively low percentage of amino acid residues in the disallowed regions suggested that the 3-D structure of CamCPR was validated.

### Quantitative analysis of tissue-specific expression of *CamCPR*

**Fig 6:**
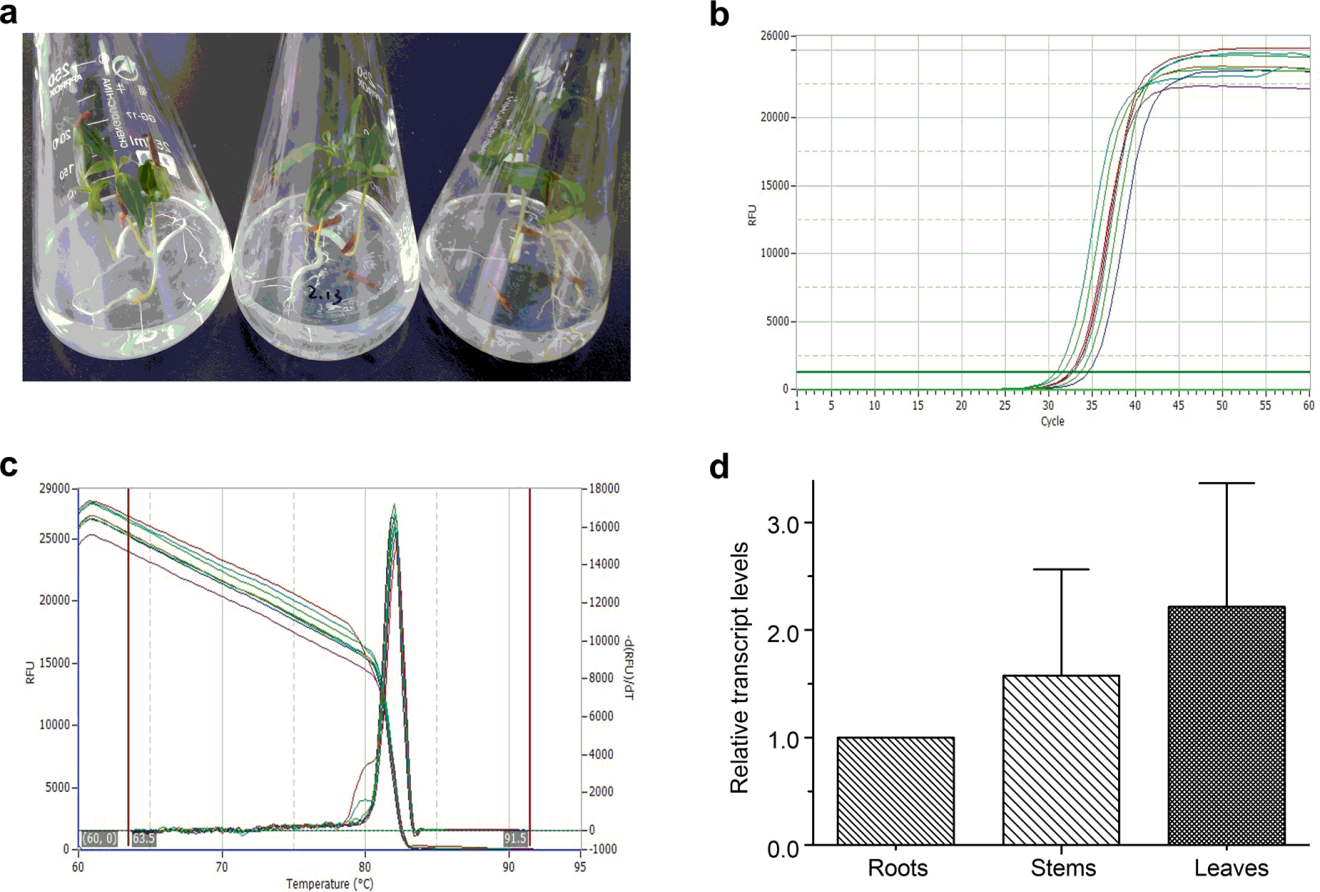
Quantitative real-time PCR of *CamCPR*. a, the 20-day-old *C*. *acuminata* seedlings; b, the real-time PCR amplification curves of *CamCPR* using rtCamCPR-F and rtCamCPR-R primers (Table 1); c, the PCR products melting curves of *CamCPR*; and d, the relative transcript levels of *CamCPR* in different tissues. The relative transcript level of *CamCPR* in the roots was set as control. Values are reported as means with standard error bars of three independent biological samples.

The real-time PCR amplification of *CamCPR* was performed to evaluate its expression in different tissues (Fig 6). The results showed that *CamCPR* was expressed in all tissues of *C*. *auminata* seedlings. However, the transcript level of *CamCPR* in stems was 1.5-fold higher than that of the roots. The leaves showed 2.2-fold higher transcript level than the roots.

## Discussion

**Fig 7:**
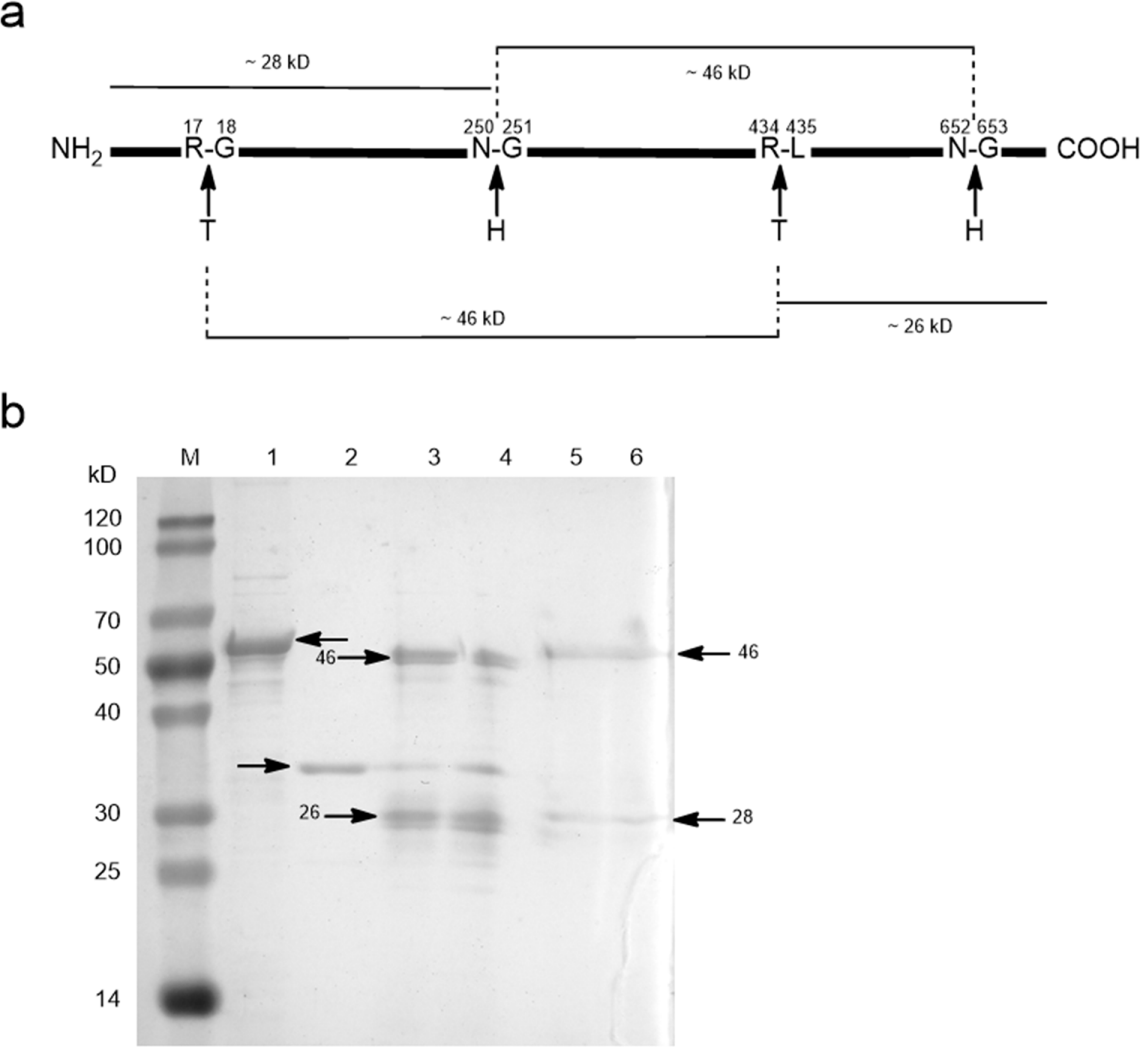
Amino acid residues alignment of CamCPR, caa_locus_6894, caa_locus_12198, and caa_locus_112450 using Clustal Omega multiple aligment tool. The identical amino acid residues between CamCPR and caa_locus_6894 were highlighted in red, between CamCPR and caa_locus_12198 were in green, and between CamCPR and caa_locus_112450 were in blue.

CPRs, the reduction partner of eukaryotic cytochrome P450s, transfer electrons from electron donors such as NADPH to the central heme iron of P450s to support their oxidation reactions [22]. CPRs and P450s constitute multicomponent redox enzyme systems [33], which plays a pivotal role in the growth, development, and metabolism of eukaryotic organisms [26]. Generally speaking, each enzymatic oxidation reaction is catalyzed by a special P450 enzyme, which means that there are many P450s presented in eukaryotic organisms. Many CPRs have been identified and functionally characterized from living organisms. It was reported that only one CPR encoding gene presented in yeasts and animals [24,29,30]. The CPR serves as a versatile redox partner to communicate with different P450s and support P450s function in yeasts and animals. Meanwhile plants contain one, two or three paralogs of CPRs with different molecular weights, subcellular localizations and regulatory mechanisms [23,24,25,26,27,28]. Each plant has at least one constitutively expressed CPR to support its growth, development, and metabolism. Some species have inducible CPR that was suggested to support the plants to defend against environmental stresses. For the plants that have only one CPR encoding gene, it was suggested that the CPR is also involved in other biological processes. For *C*. *acuminata*, one CPR was obtained in this study using the homology cloning strategy, which was consistent with the transcriptome data analysis of *C*. *acuminata* (Fig 7). Four gene fragments encoding putative CPRs, caa_locus_6894, caa_locus_12198, caa_locus_112450, and caa_locus_37170, were retrieved from the Medicinal Plant Genomics Resource consortium (http://medicinalplantgenomics.msu.edu). These gene fragments were deduced to encode 187-, 94-, 58-, and 39-amino acid residues, respectively. The deduced amino acid residue sequence of each putative CPRs was set as query to search NCBI using BLAST program. The caa_locus_37170 was ruled out because it was annotated as a hypothetic protein. The multiple amino acid residues alignment of CamCPR, caa_locus_6894, caa_locus_12198, and caa_locus_112450 showed that the deduced amino acid residues from the transcriptome sequencing are part of CamCPR (Fig 7). The three putative CPRs segments, caa_locus_6894, caa_locus_12198, and caa_locus_112450, were suggested to be presented in young bark, immature leaf, callus, root culture, young flower, immature fruit, mature fruit, leaf, cotylendones, whole seedlings, young leaf, mature leaf, immature bark, and entire root, which was consistent with the real-time quantitative analysis of *CamCPR* expression. The mRNA of *CamCPR* was detected in all tissues of *C*. *acuminata* seedlings, indicating that the CamCPR is constitutively expressed in *C*. *acuminata* to support P450s oxidation reactions.

It was found that the interacting domains of P450s and CPRs are highly conserved [34]. These domains were presented in CamCPR (Fig 2), which was evidenced by its cytochrome c reducing activity and its electrons supporting to CYP73A25, a functional P450 from cotton, to hydroxylate cinnamic acid to form 4-hydroxycinnamic acid. CPRs from vascular plants were clustered into two major phylogenetic groups based on the N-terminal hydrophobic regions [25]. CamCPR was categorized in group II CPRs, neighboring with AtCPR2, CrCPR and NfCPR1 (Fig 3).

**Fig 8:**
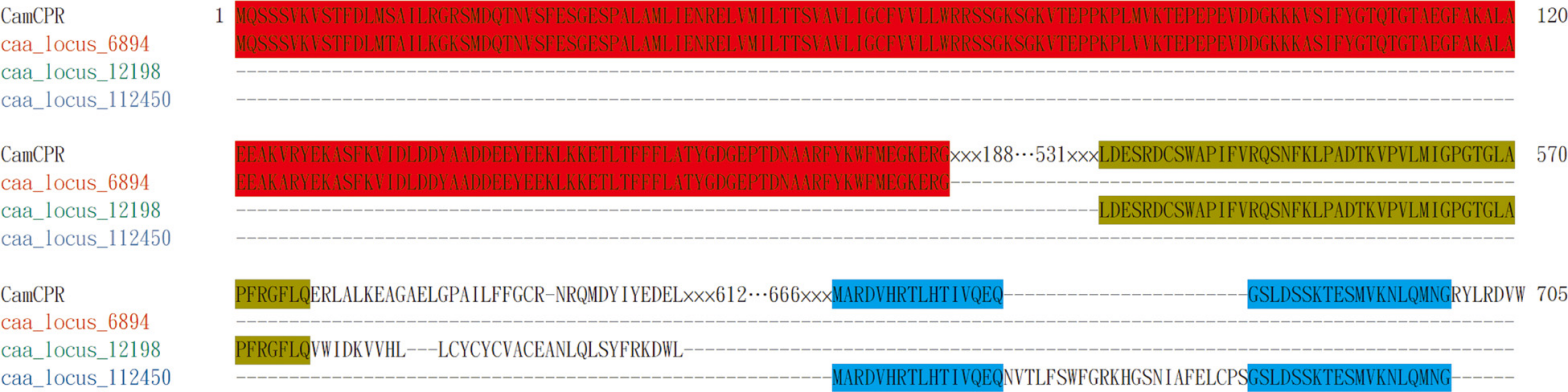
Enzymatic digestion and chemical degradation of tCamCPR. The predicted cleavage sites of tCamCPR (a) and the detected peptide fragments by SDS-PAGE analysis (b). T, thrombin; H, Hydroxylamine; M, protein ruler; Lane 1, tCamCPR; Lane 2, thrombin; Enzyme digestion products of tCamCPR with 1 U / 50 μL (Lane 3) and 2 U / 50 μL (Lane 4) of thrombin were used [37]; Lanes 5 and 6, chemical degradation products of tCamCPR by hydroxylamine [38].

Tryptophan is the last C-terminal amino acid residue of most CPRs, for instance, W705 in CamCPR (Fig 2a). The tryptophan located in the nicotinamide binding site of CPRs, which plays a very important role in the discrimination between NADPH and NADH [35,36]. This amino acid residue serves as a trigger for releasing oxidized nicotinamide [35,36]. The intact and truncate CamCPRs with N- or C-terminal His_6_-tag were overexpressed and purified to homogeneity. The cytochrome c reducing activity assays showed that the recombinant protein with free last tryptophan residue showed 18- ~ 30-fold higher reduction activity than the fusion enzymes with C-terminal His_6_-tag (Table 2), which supported a previous conclusion that the free tryptophan is highly conserved and very important in CPRs, together with those mutation experiments [32,33]. It should be noted that the observed molecular weight of tCamCPR from SDS-PAGE analysis (panel II, Fig 4a) is smaller than its theoretical molecular weight. However the tCamCPR can be separated by Ni-NTA affinity resin, indicating an entire N-terminus of tCamCPR. Also the functional characterization suggested that the last tryptophan (W660 of tCamCPR) is present in tCamCPR. Enzymatic digestion and chemical degradation of tCamCPR were performed to confirm it (Fig 8). Thrombin was used to digest tCamCPR, according to the reported procedure [37]. Two main peptide fragments with ~ 46 and 26 kD were predicted (Fig 8a) and detected by SDS-PAGE analysis (Lanes 3 and 4, Fig 8b). The amide bond between Asn and Gly can be cleaved selectively by hydroxylamine [38]. Two cleavage sites were present in tCamCPR (Fig 8a) and the two predicted peptide fragments with ~ 28 and 46 kD (Fig 8a) were also detected by SDS-PAGE analysis (Lanes 5 and 6, Fig 8b). The results showed that tCamCPR is intact. Perhaps the abnormal gel shifting of tCamCPR is due to its amino acid residue composition [39].

Metabolic engineering is a highly efficient alternative for production of pharmaceutically important natural products in heterologous hosts via biotechnology such as microbial fermentation. However, an important prerequisite for any attempt at metabolic engineering is the detailed knowledge of the underlying biosynthetic and regulatory pathways in plants [27]. The present study and the other previous reports have shown that CPRs are highly conserved and CPRs from different plants can at least partially support the oxidation ability of P450s from different species [36]. According to the ‘‘share your parts” principle [36], CamCPR can be used as an ideal bio-brick in synthetic biology approaches to re-design or develop entirely different combinations of existing biological systems to produce CAM.

## Conclusion

In summary, an NADPH-cytochrome P450 reductase CamCPR encoding gene was cloned from *C*. *acuminata*. CamCPR showed 70 ~ 85% identities to other characterized plant CPRs. The intact and truncate CamCPR with N- or C- His_6_-tag were overexpressed in *E*. *coli* and purified to homogeneity. CamCPRs showed NADPH-dependent reductase activity toward chemical and protein substrates. CamCPR can support the oxidation activity of a heterogenous P450 from cotton. The characterization and identification of CamCPR not only extends the plant CPRs family, but also facilitates the future functional studies of the enzyme interacting with other components of the cytochrome P450 enzyme systems. Additionally, the cloning and functional characterization of CamCPR will be helpful to uncover the mysterious mechanisms of the biosynthesis of CAM. The present study established a platform to characterize the P450 enzymes involved in the development, growth, and metabolism of plants
