# Correction: Molecular Cloning, Heterologous Expression, and Functional Characterization of an NADPH-Cytochrome P450 Reductase Gene from *Camptotheca acuminata*, a Camptothecin-Producing Plant

**DOI:** 10.1371/journal.pone.0139647

**Published:** 2015-09-25

**Authors:** Xixing Qu, Xiang Pu, Fei Chen, Yun Yang, Lixia Yang, Guolin Zhang, Yinggang Luo

The images for Figs 7 and 8 are incorrectly switched. The image that appears as Fig 7 should be Fig 8, and the image that appears as Fig 8 should be Fig 7. The figure captions appear in the correct order. Please see the corrected Fig 7 here.

**Fig 7:**
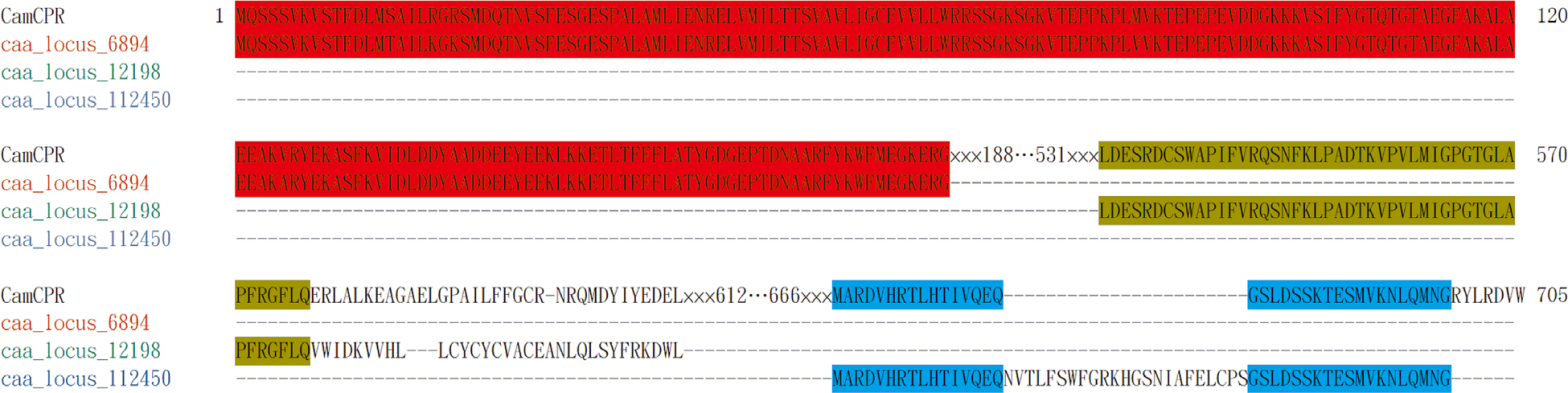
Amino acid residues alignment of CamCPR, caa_locus_6894, caa_locus_12198, and caa_locus_112450 using Clustal Omega multiple aligment tool. The identical amino acid residues between CamCPR and caa_locus_6894 were highlighted in red, between CamCPR and caa_locus_12198 were in green, and between CamCPR and caa_locus_112450 were in blue.

Please see the corrected Fig 8 here.

**Fig 8:**
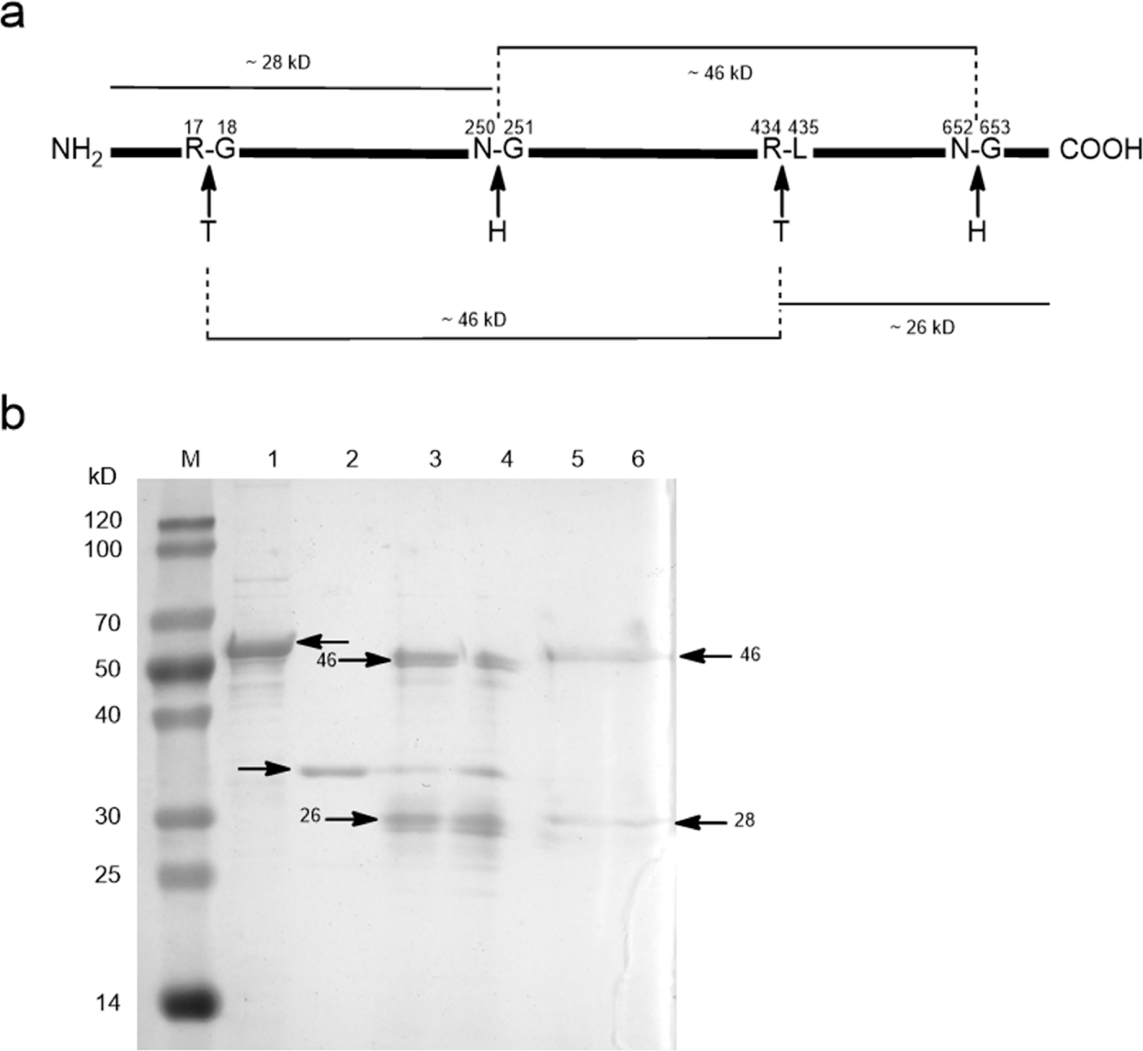
Enzymatic digestion and chemical degradation of tCamCPR. The predicted cleavage sites of tCamCPR (a) and the detected peptide fragments by SDS-PAGE analysis (b). T, thrombin; H, Hydroxylamine; M, protein ruler; Lane 1, tCamCPR; Lane 2, thrombin; Enzyme digestion products of tCamCPR with 1 U / 50 μL (Lane 3) and 2 U / 50 μL (Lane 4) of thrombin were used [37]; Lanes 5 and 6, chemical degradation products of tCamCPR by hydroxylamine [38].
